# The Effect of Lung Cancer on Cytokine Expression in Peripheral Blood Mononuclear Cells

**DOI:** 10.1371/journal.pone.0064456

**Published:** 2013-06-06

**Authors:** David H. Chang, John R. Rutledge, Ankur A. Patel, Barbara G. Heerdt, Leonard H. Augenlicht, Robert J. Korst

**Affiliations:** 1 Center for Cancer Research and Genomic Medicine, The Daniel and Gloria Blumenthal Cancer Center, Paramus, New Jersey, United States of America; 2 Division of Thoracic Surgery, Department of Surgery, The Valley Hospital, Ridgewood, New Jersey, United States of America; 3 Albert Einstein Cancer Center, Montefiore Medical Center, Bronx, New York, New York, United States of America; Roswell Park Cancer Institute, United States of America

## Abstract

The purpose of this study is to evaluate cytokine expression by peripheral blood mononuclear cells (PBMC) from stage I lung cancer patients and to confirm these expression patterns by exposing PBMCs to lung cancer cells *in vitro*. Five altered cytokines in stage I lung cancer patients (CCL3, IL8, IL1β, CXCL10, sIL2Rα) were identified in plasma from subjects (n = 15) before and after resection using a 30-plex panel protein assay. Gene expression studies using quantitative RT-qPCR were performed on PBMCs from stage I lung cancer patients (n = 62) before and after resection, and compared to non-cancer patients (n = 32) before and after surgery for benign disease. Co-culture experiments that exposed healthy donor PBMCs to lung cancer cells *in vitro* were performed to evaluate the effect on PBMC cytokine expression. PBMC gene expression of CCL3, IL8 and IL1β was higher in lung cancer patients compared to the same patients at each of four sequential timepoints after removal of their tumors, while CXCL10 and IL2Rα were essentially unchanged. This pattern was also detected when lung cancer patients were compared to non-cancer patients. When non-cancer patients underwent surgery for benign diseases, these cytokine expression changes were not demonstrable. Lung cancer cell lines, but not benign bronchial epithelial cells, induced similar changes in cytokine gene and protein expression by healthy donor PBMCs in an *in vitro* co-culture system. We conclude that PBMCs from stage I lung cancer patients possess distinct cytokine expression patterns compared to both non-cancer patients, and lung cancer patients following tumor removal. These expression patterns are replicated by healthy donor PBMCs exposed to lung cancer cell lines, but not benign bronchial epithelial cells *in vitro*. These findings have implications for understanding the immune response to lung cancer.

## Introduction

Lung cancer remains the leading cause of cancer-related mortality in the United States, and is expected to account for more deaths in 2012 than colorectal, breast, and prostate cancers combined [1]. Although lung cancer has been considered to be a poorly immunogenic malignancy, several lines of evidence suggest an influence of the host’s immune system in altering lung cancer development and progression. These include reports of enhanced survival in lung cancer patients with postoperative infections [2], increased recurrence rates among patients who receive perioperative blood transfusions [3], [4], and higher incidence rates among immunosuppressed individuals such as those who test positive for the Human Immunodeficiency Virus (HIV) [5]. Despite these observations, the role of the host immune system in the development and progression of lung cancer remains unclear.

Cytokines and chemokines (chemotactic cytokines) are important endogenous regulators of the immune system and are secreted by immune cells, as well as by tumor. In the cancer patient, these molecules possess diverse and complex roles in the function and trafficking of immune and other cells, including endothelial progenitor cells [6]. Cytokine and chemokine networks can also be “hijacked” by cancer cells to provide an environment conducive to tumor growth [6], [7].

Given the unclear role of antitumor immunity in lung cancer patients, combined with the global importance of cytokines and chemokines to the immune response, we hypothesized that lung cancer would have specific effects on cytokine/chemokine expression and secretion by peripheral blood mononuclear cells (PBMC). In this regard, the purpose of the present study is threefold. First, to determine if patients with stage I lung cancer exhibit altered cytokine expression in circulating plasma and PBMCs prior to potentially curative resection, compared to after resection, and to identify differentially expressed cytokines. Second, to confirm this differential expression in PBMCs obtained from a larger cohort of stage I lung cancer patients, and compare these results to a cohort of patients undergoing thoracic surgery for benign diseases. Third, to determine if this differential cytokine expression is triggered when healthy donor PBMCs are exposed to lung cancer cells *in vitro*.

## Materials and Methods

### Patient Population

This study was reviewed and approved by The Valley Hospital Institutional Review Board and written informed consent was obtained from all study participants. Two groups of patients were investigated. The lung cancer patient group included individuals undergoing curative pulmonary lobectomy and mediastinal lymph node dissection, with stage I status confirmed pathologically. The control patient group included non-cancer patients undergoing thoracic surgery for benign conditions, such as spontaneous pneumothorax, diagnostic resection of a benign lung nodule, bullous lung disease, bronchiectasis, mediastinal/pulmonary cysts, and hiatal hernia repair.

Patients were excluded from the study if they were receiving chronic steroid therapy, found to have advanced lung cancer (stage II, III, IV) pathologically after resection, had other concomitant cancers other than non-melanoma skin cancer, required adjuvant or neoadjuvant chemotherapy, radiotherapy or both, or had a history of HIV infection, hepatitis B or C or other immunosuppressive or myeloproliferative illnesses. Control patients were excluded if they were undergoing thoracic surgery for inflammatory/infectious conditions (empyema, interstitial lung disease, or active pneumonitis) or had a history of other types of cancer.

Preoperative blood samples were collected during a two week window prior to surgery, whereas postoperative samples were obtained at 3 (2–4 months), 6 (5–7 months), 9 (8–10 months) and 12 (11–13 months) after surgery unless otherwise stated.

### Blood Specimen Collection and Processing

Lung cancer and control patient blood was drawn by peripheral venipuncture into BD Vacutainer CPT Cell Preparation Tubes with Sodium Heparin (Becton Dickinson, Franklin Lakes, NJ). Blood processing was done according to the manufacturer’s protocol. PBMC pellets were harvested and stored at −80°C for RNA extraction and subsequent assays. Plasma was also saved and stored at −80°C.

Healthy donor blood leukocytes (buffy coat) used in the *in vitro* experiments were purchased from the Community Blood Services (Paramus, NJ). These PBMCs were prepared by centrifugation of blood on Ficoll-Paque PLUS density gradient medium (GE Healthcare, Pittsburgh, PA) for 30 minutes at 805×*g* rcf (relative centrifugal force) at room temperature. Isolated PBMCs were retrieved and washed twice in PBS by centrifugation for 15 and 10 min at 453×*g* at 4°C. Isolated PBMCs were then used for the *in vitro* co-culture assays.

### Luminex Assay

The Luminex assay is a multiplex bead-based immunoassay, which allows for the simultaneous measurement of multiple analytes (e.g. cytokines) using a library of antibody-coupled color-coded beads (called microspheres). For the initial screening of plasma proteins, plasma was collected from 15 stage I lung cancer patients preoperatively and 3–7 months postoperatively. Plasma samples were then analyzed using the Cytokine Human 30-plex Panel, LHC6003, (Luminex platform; Life Technologies; Carlsbad, CA). Assays were performed according to the manufacturer’s protocol with the exception that samples, beads and buffer were incubated overnight at 4°C. Reactions were performed using the Luminex 100 instrument (Luminex, Austin, TX), standard curves were generated, and data were collected and analyzed using Luminex 100 Integrated Software version 2.3. Supernatants from the *in vitro* co-culture experiments were also evaluated using the Luminex assay, according to the protocol described above. For the initial plasma screening studies, relative protein levels were expressed by calculating the ratio of the mean fold change in protein expression (preoperatively to postoperatively), and transforming the values to the log_2_ scale. Patients with at least one data point exhibiting expression above the Luminex minimal detection level were included in the ratio calculations.

### PBMC Gene Expression Database

Gene expression in PBMC of six lung cancer cases, before and 2–5 months after curative resection was obtained using Affymetrix U133+2.0 microarrays, using RNA isolated from PBMC cell lysates as opposed to plasma samples. This work has been described previously [8]. Hybridization intensities were used to compute fold differences between preoperative and postoperative samples.

### RNA Extraction and Quality Assessment

RNA was extracted from PBMC lysates using RNeasy Mini Kit (Qiagen Sciences, Valencia, CA) according to the manufacturer’s protocol with the following modifications. Frozen PBMC pellets were lysed directly in RLT buffer to minimize RNA degradation. Cell lysates were passed through QIAshredder (Qiagen) prior to subsequent purification as described in the protocol. Ribonculease-free DNase (Qiagen) was used during on-column digestion of DNA to minimize the presence of residual genomic DNA.

RNA quality and concentration were assessed using RNA Nano chips with Agilent 2100 Bioanalyzer (Agilent Technologies, Santa Clara, CA), and analyzed using the 2100 Expert Software (Agilent). The RIN (RNA Integrity Number) for the extracted RNA was in the range of 7.0 to 10.0 with the mean of 8.8±0.68 for all RNA samples used in this study.

### Real-time Quantitative Reverse Transcription PCR (RT-qPCR)

The RT-qPCR experiments were performed using the 7500 Real Time PCR instrument from Applied Biosystems (Life Technologies Corp., Carlsbad, CA). All materials and protocols were obtained from Applied Biosystems. Primer and probe sets (**Table S1**) were designed using Primer Express Software (Applied Biosystems) following the standard set of design criteria established by the software. Sets spanned an intron and bridged an exon-exon junction and were made to specification by either Applied Biosystems or Sigma-Aldrich (St. Louis, MO). Primers and probes were tested empirically for the absence of interference before being used for multiplexing. The normalizing control used for all RT-qPCR experiments was β-Actin (gene symbol ACTB), which was used as the reference gene for all calculations. RT-qPCR multiplex assays consisting of three to four sets of primers and probes (including that for ACTB.) were performed [9].

The RT reaction was performed with 2 µg of RNA per reaction using High Capacity RNA to cDNA kit (Life Technologies Corp., Carlsbad, CA). Quantitative PCR reaction was performed using 30 ng of cDNA in triplicate. Fold change calculations were performed using the “comparative C(T) method” established by the manufacturer [10]. C(T), cycle threshold, is also referred to as Cq (Quantification cycle) value. For calculation, the average of the triplicate Cq values was used for analysis. Cq values were normalized to those for ACTB.

### Cell Culture and *in vitro* Co-culture Experiment

The A549 and HCC827 human lung cancer cell lines were purchased from ATCC (Manassas, VA). A549 cells were cultured in F12K medium, HCC827 cells in RPMI. Both culture media were supplemented with 10% fetal bovine serum, 0.028% HEPES (4-(2-hydroxyethyl)-1-piperazineethanesulfonic acid), 0.02% Sodium Pyruvate, 0.02% Penicillin/Streptomycin, and 0.004% Gentamicin. All reagents used for cell culture were purchased from Life Technologies (Carlsbad, CA). The primary human bronchial epithelial cells (NHBE), corresponding BEBM culture medium (supplemented with BEGM bullet kit), and reagents were purchased from Lonza (Allendale, NJ). NHBE culture and subculture was performed according to the vendor’s protocol (Lonza). Cell cultures were maintained at 37°C with 5% CO_2_.

A549, HCC927 and NHBE cells were grown for two days in 3 ml medium in 35 mm (6-well) dishes (BD Biosciences, Franklin Lakes, NJ) to 80–90% confluence. After two days, 0.4-µm transwell inserts (Corning Corp, Corning, NY) were placed into the wells and 5×10^6^ of healthy donor PBMCs, suspended in 3 ml of corresponding cell culture medium, were added to each transwell. Co-culture controls consisted of wells containing only complete cell culture media (F12K, RPMI or BEBM, supplemented as described above) without the malignant or benign epithelial cells. Co-cultures were then incubated for 6 and 18 hours. Following incubation, cell culture supernatants were collected, centrifuged (453×*g* rcf, 4°C, 15 min), and aliquots stored at −80°C for the Luminex assay. For gene expression analysis, the transwell inserts were removed and PBMCs immediately lysed *in situ* using RLT buffer (RNeasy kit; Qiagen) and either stored at −80°C or used immediately for RNA extraction for RT-qPCR. For flow cytometric analysis of protein expression, 10 hours after adding PBMCs to the transwell inserts, 1 µl/ml of Golgi-Plug/Brefeldin A (BD Biosciences) was added to inhibit protein transport. PMA (phorbol-12-myristate-13-acetate, Sigma-Aldrich, St. Louis, MO) was added to selected wells as a positive control at the same time with GolgiPlug. After 8 more hours of incubation, PBMCs were harvested and used for flow cytometry to detect intracellular cytokines.

### Flow Cytometry

To define PBMC cell populations, patient blood was drawn into BD Vacutainer Plus Plastic K_2_EDTA at the same time it was collected for RNA extraction. Immune cell populations were characterized by flow cytometry following the standard operating procedures established by The Valley Hospital Clinical Pathology Laboratory. Flow Cytometry was performed using a Beckman Coulter Cytomics FC500 and CXP cytometer software. Data analysis was accomplished using Kaluza software from Beckman Coulter. The reagents for flow cytometry were purchased from various sources: Anti-human-CD11b-PECF594, anti-human-IL8-PE, anti-human-CD14-FITC, and anti-human-IL1β-PE from BD Biosciences; anti-human-CD8-PE-Cy5, anti-human-CD56-PE-Cy7, and anti-human-CCL3-PE from eBioscience (San Diego, CA); anti-human-CD3-ECD and anti-human-CD45-PE-Cy7 from Beckman Coulter (Brea, CA); anti-human-CD4-FITC and anti-human-CD19-PE-Cy5 from Dako (Carpinteria, CA).

For the *in vitro* co-culture experiments, intracellular cytokine staining was performed following the Cytofix/Cytoperm protocol from BD Biosciences. Briefly, PBMCs from the co-culture experiments were harvested, washed and stained for cell surface markers. After surface staining, cells were washed, fixed with Cytofix/Cytoperm and stained for intracellular CCL3, IL8, and IL1β. After the staining, cells were washed again prior to analyses.

### Statistical Analysis

Differences in mean expression levels between the cancer patients and control patients and changes in mean expression levels over the course of time were evaluated concurrently using repeated measures analysis of variance (ANOVA). This type of ANOVA accounts for the simultaneous effect of both factors on expression [11]. The comparison of the cancer patients and control patients essentially evaluates the effect of tumor presence on expression levels, while the assessment of expression as a function of time evaluates the effect that undergoing a surgical procedure had on each patient in the study.

P values ≤0.05 were considered statistically significant. IBM-SPSS Statistics software (version 19) was used for all statistical analyses.

## Results

### Initial Analysis of Plasma from Stage I Lung Cancer Patients for Cytokine Protein Levels

To assess whether cytokines and chemokines were differentially expressed in the plasma from stage I lung cancer patients (n = 15) before and after (3–7 months post-operatively) curative resection a human multiplex immunoassay was used, and the results are displayed in **Table 1**. Of the original 30 immune cytokine/chemokine/growth factor targets assayed by Luminex, levels of 21 targets were above the detection limit in the lung cancer patients’ plasma samples (**Table 1**), while expression levels of 9 targets (Tumor Necrosis Factor alpha, IL2, IL4, IL5, IL10, IL13, IL17, Granulocyte Macrophage-Colony Stimulating Factor, Interferon gamma) were below the detection limit in both the preoperative and postoperative samples (data not shown).

**Table 1:** Post-operative changes in plasma protein and PBMC mRNA levels of cytokines.

	Plasma Protein				PBMC mRNA			
	Preop/postop (n = 15)				Preop/postop (n = 6)			
	Mean	SD	Range	P value	Mean	SD	Range	P value
**CCL3**	**1.28**	**1.55**	**(0.63–3.34)**	**0.11**	**1.45**	**0.78**	**(0.12–2.50)**	**<0.0001**
**IL8**	**0.80**	**0.88**	**(**−**0.54–2.95)**	**0.31**	**1.80**	**0.61**	**(0.61–2.49)**	**<0.0001**
IL6	0.52	0.57	(−0.82–0.72)	0.50	0.42	1.56	(−2.18–3.12)	0.44
**IL1β**	**0.40**	**0.30**	**(**−**0.32–0.52)**	**0.09**	**1.20**	**1.27**	**(**−**0.28–2.98)**	**0.02**
EGF	0.40	0.87	(−1.06–1.63)	0.50	−0.58	1.28	(−2.52–0.89)	0.21
IL7	0.33	0.40	(−0.43–0.68)	0.74	0.03	0.89	(−1.54–1.14)	0.92
CCL5	0.29	1.14	(−1.41–2.33)	0.42	−0.67	1.00	(−1.88–0.46)	0.08
FGFβ	0.28	0.82	(−0.26–1.67)	0.27	−0.74	0.91	(−1.89–0.65)	0.04
IL12	0.20	0.51	(−0.43–1.55)	0.37	0.00	0.57	(−0.88–0.99)	0.99
CCL11	0.15	0.47	(−0.41–1.13)	1.00	0.73	1.46	(−1.12–2.54)	0.17
IL1Rα	0.15	0.34	(−0.67–0.77)	0.08	0.33	0.98	(−1.25–1.81)	0.33
IFNα	0.10	0.56	(−0.95–0.70)	0.92	0.32	1.19	(−1.16–2.37)	0.44
VEGF	−0.01	0.23	(−0.22–0.37)	0.83	1.28	0.78	(0.03–2.34)	<0.0001
CCL2	−0.03	0.56	(−1.24–0.85)	0.86	−0.59	1.00	(−2.09–0.94)	0.12
G-CSF	−0.03	0.42	(−0.99–0.61)	0.68	0.59	1.66	(−1.67–2.70)	0.32
CXCL9	−0.09	0.88	(−1.71–1.60)	0.14	1.37	0.63	(0.75–2.84)	<0.0001
CCL4	−0.12	1.01	(−1.88–1.72)	0.29	0.83	1.07	(−0.37–2.93)	0.05
IL15	−0.16	0.41	(−0.50–0.67)	0.25	0.21	0.57	(−0.55–1.37)	0.31
HGF	−0.31	0.65	(−1.56–0.91)	0.97	0.40	1.14	(−1.32–1.83)	0.33
**CXCL10**	−**0.74**	**1.21**	**(**−**2.58–2.32)**	**0.16**	−**0.11**	**1.04**	**(**−**1.72–1.23)**	**0.76**
**IL2Rα**	−**0.77**	**0.60**	**(**−**1.76–** −**0.19)**	**0.04**	**0.05**	**1.29**	**(**−**1.47–2.89)**	**0.91**

Results of initial screening of plasma for cytokine/chemokine/growth factor levels and comparison to PBMC gene expression (microarray) database are displayed. Mean ratios of preoperative expression over postoperative expression are presented along with their corresponding standard deviations and ranges. A positive value indicates higher levels preoperatively. All values are expressed using a log_2_ scale. P-values were obtained using paired t-tests. The targets are ranked based on the ratios calculated for the Luminex data. The bolded cells indicate the 5 cytokines selected for further study.

### Identification of 5 genes for Further Investigation

To corroborate the initial findings for the 21 targets identified by the Luminex assay, and to help define targets for further investigation, we interrogated a microarray database previously generated in our laboratory that compares patterns of gene expression in PBMCs obtained from stage I lung cancer patients before and after curative resection. Similar to the protein data, gene expression for the majority of these 21 cytokines was higher prior to curative resection of stage I lung cancer, compared to after resection (**Table 1**).

Five cytokines were selected for further investigation by examining the initial screening plasma protein data and the microarray (mRNA) database in parallel, focusing on targets that were significantly up/downregulated (p</ = 0.05). As shown in **Table 1**, of the four cytokines (proteins) which were detected in the plasma at the highest levels *prior* to stage I lung cancer resection (CCL3 (Chemokine with CC motif ligand 3), IL8 (Interleukin 8), IL6 (Interleukin 6), IL1β (Interleukin 1 beta)), three were corroborated by the PBMC gene expression data from the microarray database (*CCL3, IL8, IL1β*) at the mRNA level. Similarly, the two cytokines (proteins) which were found at the lowest levels *prior* to stage I lung cancer resection (CXCL10 and soluble IL2Rα) were both corroborated by the PBMC gene expression data from the microarray database. **Figure S1** graphically depicts the summarized screening plasma protein results and PBMC mRNA database findings for the 5 genes selected for further study: *CCL3, IL8, IL1β, CXCL10,* and *IL2Rα*.

### Confirmation by RT-qPCR of Differentially Expressed Cytokines Following Resection of Stage I Lung Cancer

**Figure 1:**
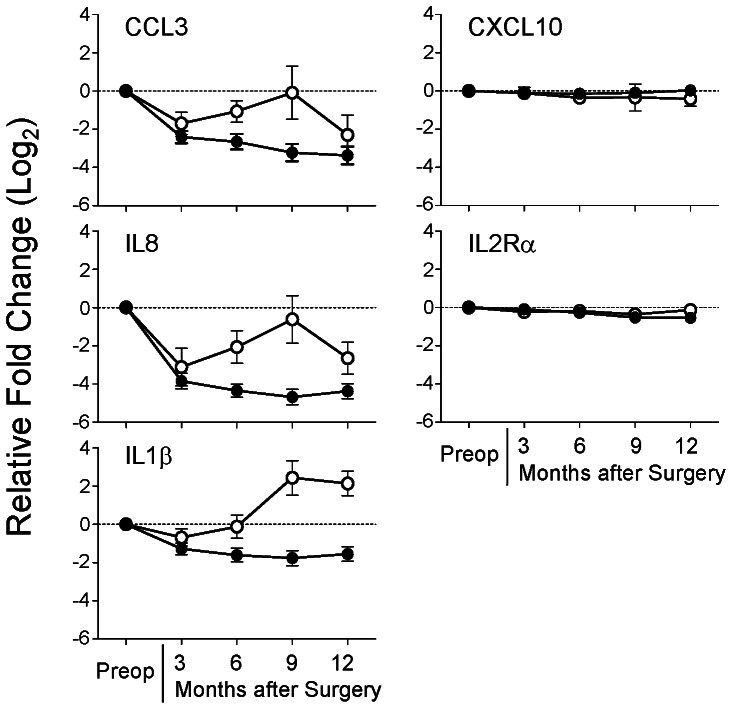
The effect of lung cancer removal on cytokine gene expression. Gene expression for 5 selected cytokines in PBMCs from stage I lung cancer (closed circles) and non-cancer control patients (open circles) after surgery are demonstrated. Each postoperative point represents the mean (+/− SEM) fold change in expression at that designated postoperative timepoint compared to the preoperative level, expressed in log_2_ scale. Preoperative values (preop) are shown as zero for reference purposes. Changes less than zero represent downregulation after surgery. For the cancer patients, the number of samples used in the calculations are: Preop: n = 58; 3 month: n = 48; 6 month: n = 43; 9 month: n = 40; 12 month: n = 40. For the control patients, the number of samples used in the calculations are: Preop: n = 16; 3 month: n = 11; 6 month: n = 9; 9 month: n = 6; 12 month: n = 7.

**Figure 2:**
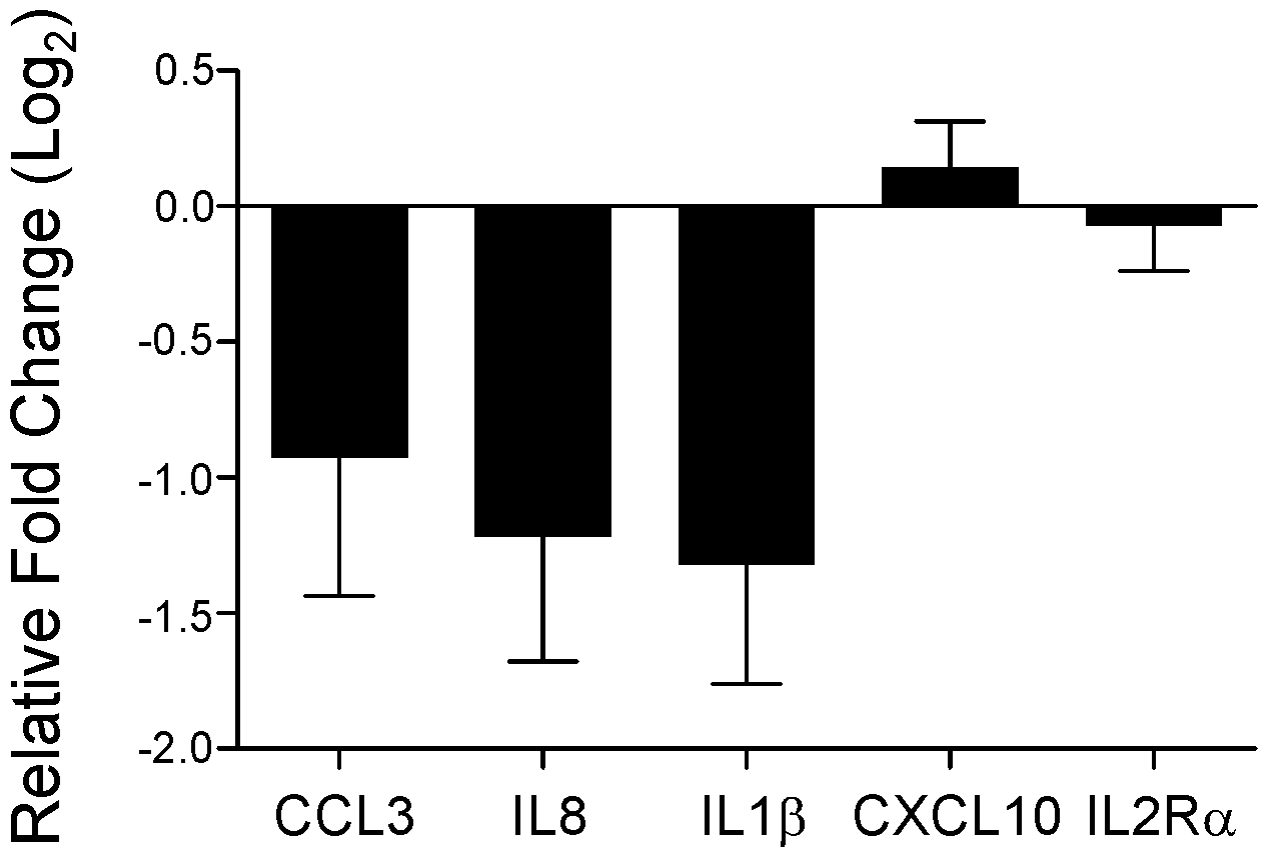
Summary of postoperative PBMC gene expression patterns for 5 selected cytokines. A repeated measures analysis of variance was used to isolate the effect of the presence or absence of lung cancer on the expression of *CCL3* (p = 0.07), *IL8* (p = 0.01), *IL1β* (p = 0.004), *CXCL10* (p = 0.4) and *IL2Rα* (p = 0.69). Each bar represents the mean (+/− SEM) relative fold change for each cytokine incorporating all postoperative data compared to the expression before surgery, expressed in log_2_ scale. A negative relative fold change indicates downregulation after stage I lung cancer resection.

To validate and extend the results obtained from the initial Luminex and microarray data, PBMC gene expression was evaluated in much larger cohorts of stage I lung cancer patients (n = 62) and control patients undergoing thoracic surgery for benign diseases (n = 32). Demographic features of these two cohorts of patients are displayed in **Table S2**.The five selected target genes, *CCL3, IL8, IL1β, CXCL10,* and *IL2Rα,* were further investigated by the real-time RT-qPCR assay. Of the 62 patients in the lung cancer cohort who underwent phlebotomy prior to resection, 58 had blood drawn for analysis during the postoperative period, as did 16 of the 32 control patients. **Figure 1** demonstrates the relative changes in expression detected for the 5 genes during the postoperative period for the lung cancer and control patients. Interestingly, PBMC gene expression for the three cytokines (*CCL3, IL8, IL1β*) detected in plasma at elevated levels *prior* to lung cancer resection during the initial screening were found to be downregulated *after* lung cancer resection at all timepoints. Further, the same pattern was not detected in the control patients undergoing thoracic surgery for benign diseases. Finally, PBMC gene expression for the 2 cytokines (*CXCL10, IL2Rα*) which were not detected in plasma at elevated levels *prior* to lung cancer resection during the initial screening were not significantly different *after* resection (**Figure 1**). Following analysis of these data using repeated measures ANOVA, it becomes clear that *IL8* and *IL1β* expression are significantly downregulated after resection of stage I lung cancer, a finding which is not associated with the effect of thoracic surgery in non-cancer patients. *CCL3* also appears to be downregulated in a similar fashion, to the extent that it approaches statistical significance (**Figure 2**). Further, flow cytometry of PBMCs shows a uniform population of lymphocyte subtypes across all patient cohorts, suggesting that changes in gene expression do not appear to be due to differences in cell representation in the PBMC lymphocyte populations, although these data do not eliminate the possibility of subtle shifts in subpopulations (**Figure S2**).

### Expression of *CCL3, IL8* and *IL1β* is Higher in PBMCs from Stage I Lung Cancer Patients Compared to Patients without Lung Cancer

**Figure 3:**
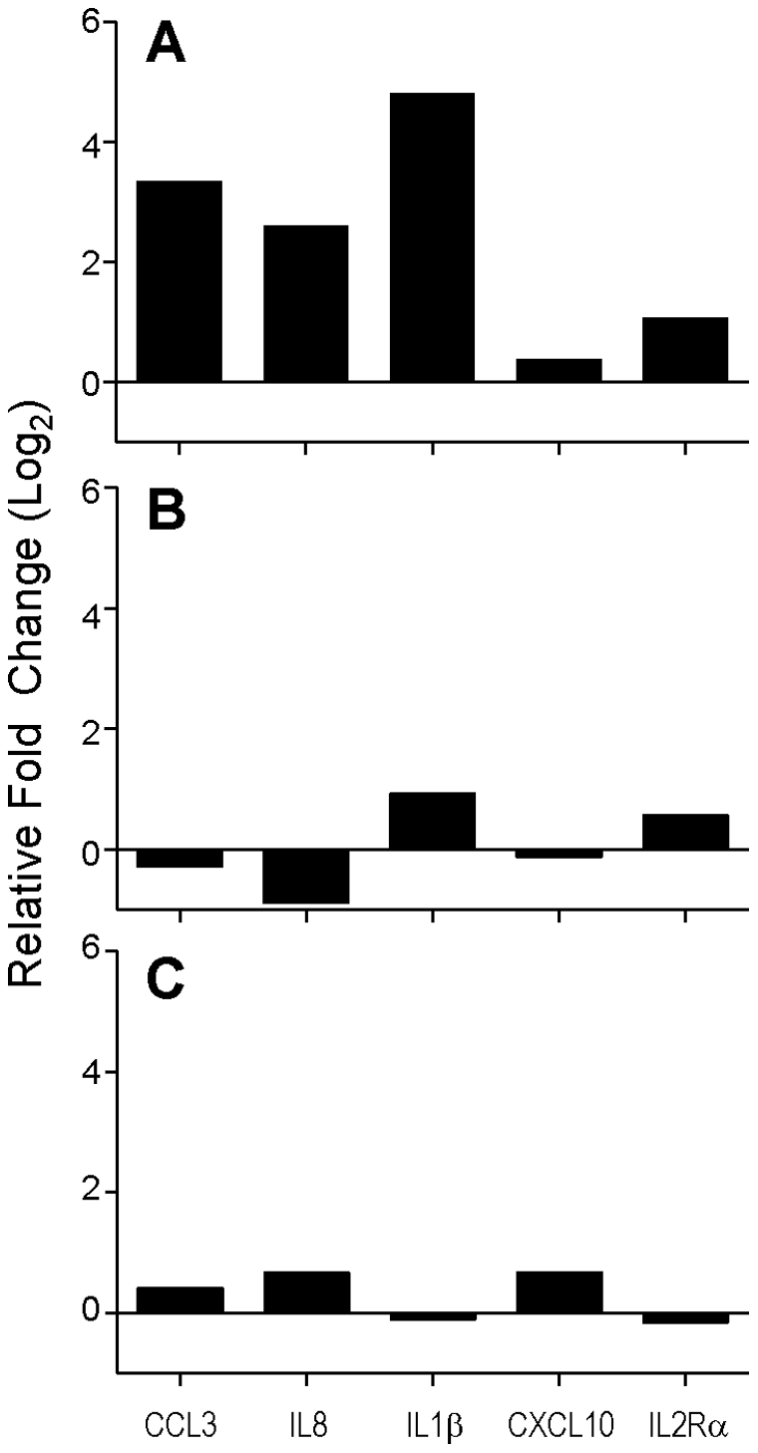
Gene expression of 5 selected cytokines in lung cancer patients compared to non-cancer patients. **A.** Preoperative PBMC gene expression in stage I lung cancer patients (n = 62) compared to non-cancer patients scheduled to undergo thoracic surgery for benign disease (n = 32). *CCL3*: p = 0.003; *IL8*: p = 0.23; *IL1β*: p = 0.05; *CXCL10*: p = 0.55; *IL2Rα*: p = 0.01. **B.** Three month postoperative PBMC gene expression in stage I lung cancer patients (n = 48) compared to non-cancer patients (n = 11). *CCL3*: p = 0.79; *IL8*: p = 0.3; *IL1β*: p = 0.1; *CXCL10*: p = 0.8; *IL2Rα*: p = 0.29. **C.** Twelve month postoperative PBMC gene expression in stage I lung cancer patients (n = 40) compared to non-cancer patients (n = 7). *CCL3*: p = 0.76; *IL8*: p = 0.52; *IL1β*: p = 0.87; *CXCL10*: p = 0.22; *IL2Rα*: p = 0.58. For all panels, each bar represents the ratio of the mean expression level in the cancer patients over the mean expression level in the control patients, expressed in log_2_ scale. A positive value indicates that expression is higher in the cancer patients compared to the control patients. Two sample t-tests were used to determine the significance of the differences in expression.

To determine if PBMCs from stage I lung cancer patients have higher expression levels of *CCL3, IL8* and *IL1β* than patients without lung cancer, the RT-qPCR results from the preoperative PBMC samples from the stage I lung cancer (n = 62) and control cohorts (n = 32) were compared. As shown in **Figure 3**, the mean expression in lung cancer patients was significantly higher relative to controls for *CCL3* and *IL1β* (10 and 29 fold, respectively). Although not statistically significant, mean expression of *IL8* was 6 fold higher in the lung cancer patients relative to controls. In contrast, although statistically significant, *IL2Rα* expression was only 2 fold higher in the lung cancer patients, while *CXCL10* expression was essentially unchanged. Importantly, at three and twelve months after surgery, expression levels appear to equalize between the cancer and control cohorts (**Figure 3**).

### The Cytokine Profile of Healthy Donor PBMCs Mimics *in vivo* Findings Following Co-culture with Lung Cancer Cell Lines

To extend analysis of the effects of lung cancer on PBMC cytokine expression, an *in vitro* co-culture system was used, as described in **Materials and Methods**. Two different human lung cancer cell lines were used in the lower transwell chambers: A549, a lung carcinoma cell line harboring a K-Ras mutation and HCC827, a lung adenocarcinoma cell line with an EGFR mutation (E746-A750 del). As a further control, benign NHBE cells were also used in the lower transwell chambers. PBMCs, used in the upper transwells, were prepared from healthy donors with no known lung cancer history.

**Figure 4:**
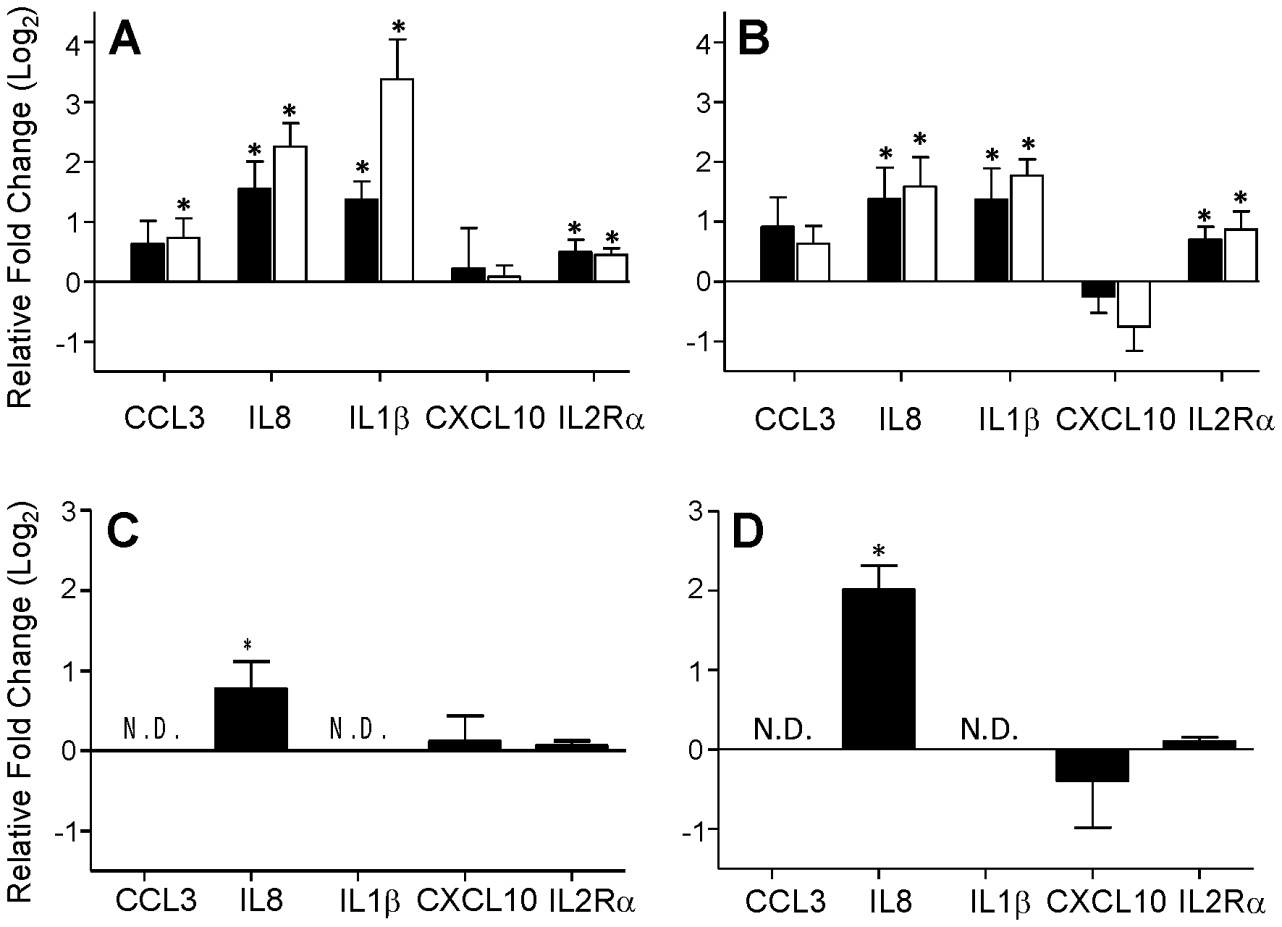
Effect human lung cancer cell lines on healthy donor PBMC cytokine expression *in vitro*. Lung cancer cell lines were co-cultured with healthy donor PBMC as described in **Materials and Methods**. PBMC gene expression and supernatant protein levels were evaluated for each of 5 selected cytokines. **A.** Gene expression in PBMC exposed to A549 cells. **B.** Gene expression in PBMC exposed to HCC827 cells. For panels **A** and **B**, each bar represents the mean (n = 8 healthy donors) relative fold change (+/− SEM) between lung cancer cell exposed PBMC and PBMC exposed to media alone, expressed in log_2_ scale. Closed bars indicate 6 hours of co-culture, while the open bars indicate 18 hours of co-culture. **C.** Cytokine protein levels in the supernatant over co-culture of A549 cells with healthy donor PBMC. **D.** Cytokine protein levels in the supernatant over co-culture of HCC827 cells with healthy donor PBMC. For panels **C** and **D**, each bar represents the mean (n = 8 healthy donors) relative fold change (+/− SEM) between lung cancer cell exposed PBMC (18 hours of co-culture) and PBMC exposed to media alone, expressed in log_2_ scale. One sample t-tests were used to determine the significance of the observed mean fold changes. In all panels, an asterisk indicates p<0.05. N.D indicates data below the detection level.

Exposure of healthy donor PBMCs for 6 and 18 hours to either A549 or HCC827 cells induced upregulation of *CCL3, IL8* and *IL1β* mRNA in a time-dependent manner when compared to sham-exposed PBMC (**Figures 4A and 4B**). Although *IL2Rα* mRNA was also upregulated, the extent is far less than that seen for *IL8* and *IL1β*. In contrast, *CXCL10* expression was either unchanged or downregulated after co-incubation with the lung cancer cell lines. Interestingly, this pattern of cytokine gene expression was not seen in the PBMC co-cultured with the benign NHBE cells (**Figure S3**). Further, the same buffy coats used for the co-culture of NHBE cells and PBMC (showing no cytokine response) were also exposed to A549 and HCC827 lung cancer cells where a response similar to that demonstrated in **Figure 4** was appreciated (data not shown).

**Figure 5:**
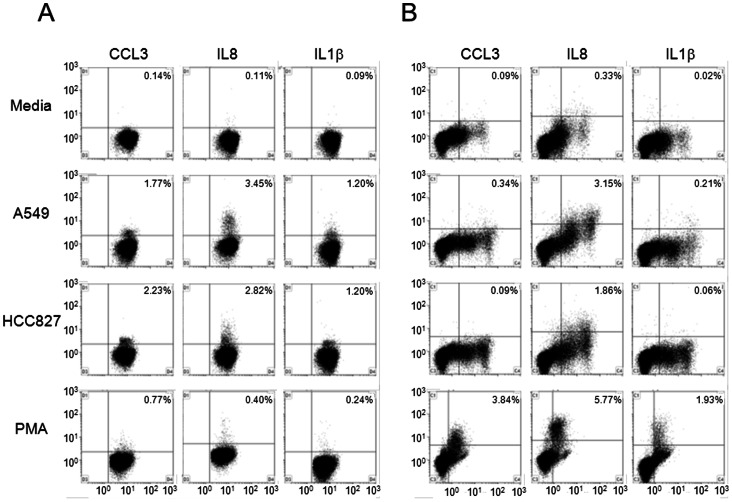
Intracellular cytokine levels in PBMC co-cultured with two lung cancer cell lines. Following co-culture, PBMC were fixed and evaluated using flow cytometry for intracellular cytokines (CCL3, IL8 and IL1β). Shown is one representative healthy donor (out of 3 performed). **A.** CD3+ cell fraction. The abscissa represents CD3 staining, while individual cytokine staining is reflected along the ordinate. The percentage of doubly stained cells is depicted in the upper right corner of each subpanel. **B.** CD14+ cell fraction. The abscissa represents CD14 staining, while individual cytokine staining is reflected along the ordinate. The percentage of doubly stained cells is depicted in the upper right corner of each subpanel.

To assess cytokine protein levels in the co-culture system, supernatants were harvested following 18 hours of co-culture with the lung cancer cell lines, and evaluated using the Luminex assay (**Figures 4C and 4D** **)**. While IL8, CXCL10 and sIL2Rα concentrations mimicked the corresponding gene expression data (**Figure 4A and 4B**), CCL3 and IL1β levels were below the limits of detection in all samples for both cell lines. To address this lack of detectable CCL3 and IL1β in the culture supernatants, PBMCs were evaluated using flow cytometry for the intracellular presence of CCL3, IL8 and IL1β. As shown in **Figure 5A**, intracellular CCL3, IL8 and IL1β were detected in the CD3+ fraction (T cells) of healthy donor PBMCs which had been co-cultured with either lung cancer cell line, but not in those cultured in the absence of lung cancer cells. A similar result was obtained for the CD14+ PBMC fraction (monocytes), shown in **Figure 5B**. Conversely, the CD19+ fraction (B cells), and CD56+ fraction (NK cells) showed no increase in the intracellular levels of these cytokines (data not shown).

## Discussion

The role of cytokines in the biology of human malignancy is complex, but much of the interaction between tumor cells and the host’s immune cells is thought to be mediated by this large family of proteins and glycoproteins [6]. As a result, there is a growing body of literature evaluating serum cytokine levels with respect to clinicopathologic features of both hematologic and solid tumors, including lung cancer. Many of these studies, however, only compared serum cytokine levels of cancer patients to those of healthy volunteers, with the emphasis placed on the potential utility of these measurements as a biomarker [12], [13]. In addition, since many cytokines can be produced by tumor cells themselves, it is not clear which cells (e.g. tumor and/or immune cells) are responsible for any aberrant cytokine levels which may be detected in the tumor bearing host. Further, uncertainty exists as to where cytokine producing cells reside in cancer patients (e.g. local tumor milieu or systemically).

Given these issues, we sought to investigate the relationship between lung cancer and circulating cytokines in greater detail. In this regard, the data demonstrate that (1) plasma levels of CCL3, IL8 and IL1β are increased in stage I lung cancer patients prior to curative resection, compared to after curative resection; (2) PBMCs obtained from stage I lung cancer patients reflect a similar pattern with regard to the expression of the genes that encode these proteins; (3) patients who undergo thoracic surgery for benign conditions do not share these patterns either before or after surgery; (4) when PBMCs from healthy donors are co-cultured with lung cancer cell lines *in vitro*, the expression of these cytokines mimics the *in vivo* pattern detected in lung cancer patients prior to tumor removal, a finding which is not reproduced when benign NHBE cells are substituted in place of the malignant cell lines.

### The Presence of Lung Cancer Alters Cytokine Expression by PBMCs *in vivo*

The initial screening of plasma for cytokine protein levels was extended by making three important comparisons. First, gene expression for 5 selected cytokine genes in PBMCs from stage I lung cancer patients was compared to the same patients following potentially curative resection. This unique approach has the advantage of using each patient as his/her own control, thereby reducing the effect of individual patient variability in cytokine expression. It is important to note, however, that approximately 30% of stage I patients will eventually develop recurrent lung cancer following curative resection, indicating that persistent disease may have been present despite resection and stage I status [14]. This recurrence rate may have contributed to variability detected in postoperative gene expression levels. Second, gene expression in PBMCs from patients with stage I lung cancer (prior to resection) were compared to a different cohort of non-cancer patients (control patients) undergoing thoracic surgery for benign disease (prior to resection). This approach was evaluated to address the potential effect of the operative procedure on cytokine gene expression, however, the results may be influenced by individual patient variation in cytokine expression. Finally, to further investigate the influence of a thoracic surgical procedure itself on cytokine gene expression, PBMC expression levels were compared in the control patients before and after surgery. Since each strategy has its limitations, by demonstrating consistent alterations in PBMC cytokine expression (or lack thereof) using this combination of approaches, the data are more compelling than if only a single approach was used. This combination of approaches is especially important because a minority of analyses from the *in vivo* experiments did not reach statistical significance as defined in **Materials and** **Methods.** However, the pattern of cytokine expression was consistent throughout all *in vivo* and *in vitro* components of this project.

Although cytokines and chemokines are generally thought to play an integral part in the communication between tumor cells and immune cells in the local tumor milieu [6], the present study suggests that circulating cells may be responsible, at least in part, for altered cytokine levels detected in the plasma/serum of patients with lung cancer. The specific PBMC fraction responsible for this altered gene expression was not evaluated *in vivo*. However, it seems likely that immune cells, as opposed to circulating tumor cells, are the most likely candidate, given the rarity (1 cancer cell per 10^8^ PBMCs) of circulating tumor cells in the bloodstream of cancer patients [15].

### The Presence of Lung Cancer Alters Cytokine Expression by Healthy Donor PBMC *in vitro*

To further investigate the influence of lung cancer on cytokine expression by PBMCs, healthy donor PBMCs were exposed to lung cancer cell lines *in vitro*, and PBMC gene as well as protein expression were evaluated. Taken together, the data suggest that healthy donor PBMCs increase their expression of CCL3, IL8 and IL1β (with little or no increases in CXCL10 and IL2Rα) when they are exposed to lung cancer cells (**Figures 4** **and** **5**). The observation that exposure of healthy donor PBMCs to a population of benign cells (**Figure S3**) does not reproduce the same result as malignant cell exposure is especially intriguing and corroborates the *in vivo* results seen in the control patients. This data supports a recent publication by Kolesar and colleagues which demonstrated increased expression of IL8 and IL1β by a monocytic cell line exposed to A549 cells *in vitro* [16]. In the present study, although IL1β and CCL3 were not detectable in the supernatants of the co-cultures (**Figure 4**), the flow cytometry data (**Figure 5**) indicates higher intracellular levels of these cytokines in specific PBMC fractions (CD3+ and CD14+ cells). Both CCL3 and IL1β are known to require posttranslational modification and the secretion of IL1β is an inefficient process, which may be responsible for the difficulty in detecting these two cytokines in the supernatants following co-culture limited to 18 hours [17], [18].

Although the data demonstrate that exposure of healthy donor PBMCs to lung cancer cell lines increases CCL3, IL8 and IL1β production by CD3+ and CD14+ PBMC fractions, it remains unclear as to the specific interaction which causes this result. Given that the small pore size of the transwell membrane used in these experiments prevents cell contact, it seems likely that a soluble mediator(s) may be involved. In addition, it is also unclear if the tumor cells themselves are secreting these cytokines in this *in vitro* system. Both of these questions require further investigation.

### Potential Significance of Altered Cytokine Expression

All three cytokines identified as upregulated in the presence of lung cancer are known to be involved in the interaction between the tumor and the host immune response. IL8 is an inflammatory cytokine associated with the promotion of neutrophil chemotaxis and degranulation [19]. It is pro-angiogenic and its expression is increased in cancer cells, endothelial cells, infiltrating neutrophils, and tumor-associated macrophages [20]. Of note, increased intratumoral expression of IL8 has been shown to have a negative impact on lung cancer patient survival [21], and higher circulating IL8 levels have correlated with progression in xenograft lung cancer models [22]. Similar to IL8, IL1β also promotes tumor progression by controlling tumor growth and invasion. IL1β induces secretion of matrix metalloproteinases as well as angiogenic factors in the tumor microenvironment [17]. Further, elevated IL1β gene expression in normal lung tissue has been linked to increased risk of developing lung cancer in an epidemiologic study [23]. CCL3, also known as Macrophage Inflammatory Protein-1 α (MIP1α), belongs to the CC chemokine family [24] and is a potent chemoattractant and activator of monocytes. In contrast to IL8 and IL1β which promote tumor growth, CCL3 possesses antitumor effects in preclinical models and has been investigated as a tumor immunotherapy strategy [25].

The findings in this study are potentially relevant in deciphering the role of cytokine expression by PBMCs in the immune response to lung cancer. Cytokines play complex roles in the cancer patient; not only do they modulate tumor growth and the immune response, but they also may be responsible for cancer-related symptoms and debilitation [26]. Thus, further elucidation of the interactions between lung tumor cells and PBMCs will be important in both the laboratory and clinical arenas. This includes the potential utility of PBMC cytokine expression as a biomarker for lung cancer. Although several existing publications have documented alterations in peripheral blood cytokine levels in lung cancer patients compared to normal individuals [2], [13], the present study interrogates this concept further by evaluating patients before and after curative resection, in addition to comparing cancer to non-cancer patients. Thus, it will be important to determine if levels of CCL3, IL8 and IL1β increase in patients who experience clinical recurrence, and thus may be biomarker of potential recurrence.

## Supporting Information

Figure S1 **Post-operative changes in plasma protein and PBMC mRNA levels of cytokines.** Graphic display of initial screening of plasma from stage I lung cancer patients before and after curative resection for the 5 selected cytokine protein levels, with corresponding PBMC gene expression data from an existing microarray database is shown. Each bar represents the ratio of the mean preoperative expression level over the mean postoperative expression level, expressed in log_2_ scale.
(TIFF)

Figure S2 **PBMC lymphocyte fractions from patients with stage I lung cancer.** PBMC pellets were subjected to flow cytometry evaluating CD3, CD4, CD8, CD19 and CD56. Each data point represents an individual blood sample, while the horizontal bars represent the mean (+/−SEM). “Pre” indicates samples obtained prior to resection.
(TIFF)

Figure S3 **Effect of benign NHBE cells on healthy donor PBMC cytokine expression** ***in vitro*** **.** NHBE cells were co-cultured with healthy donor PBMC as described in **Materials and Methods**. PBMC gene expression was evaluated for each of 5 selected cytokines. Each bar represents the mean (n = 4 healthy donors) relative fold change (+/− SEM) between NHBE cell exposed PBMC and PBMC exposed to media alone, expressed in log_2_ scale. Closed bars indicate 6 hours of co-culture, while the open bars indicate 18 hours of co-culture. N.D. indicates data below detection level. No changes were statistically significant.
(TIFF)

Table S1 **Primer and probe sets for qRT-PCR.**
(DOCX)

Table S2 **Demographic features of stage I lung cancer patients and control patients.**
(DOCX)
