# Supplementary figures and images for: The Effect of Lung Cancer on Cytokine Expression in Peripheral Blood Mononuclear Cells
Source: PLoS One. 2013 Jun 6;8(6):e64456. doi: 10.1371/journal.pone.0064456 (PMC3675097; doi:10.1371/journal.pone.0064456)

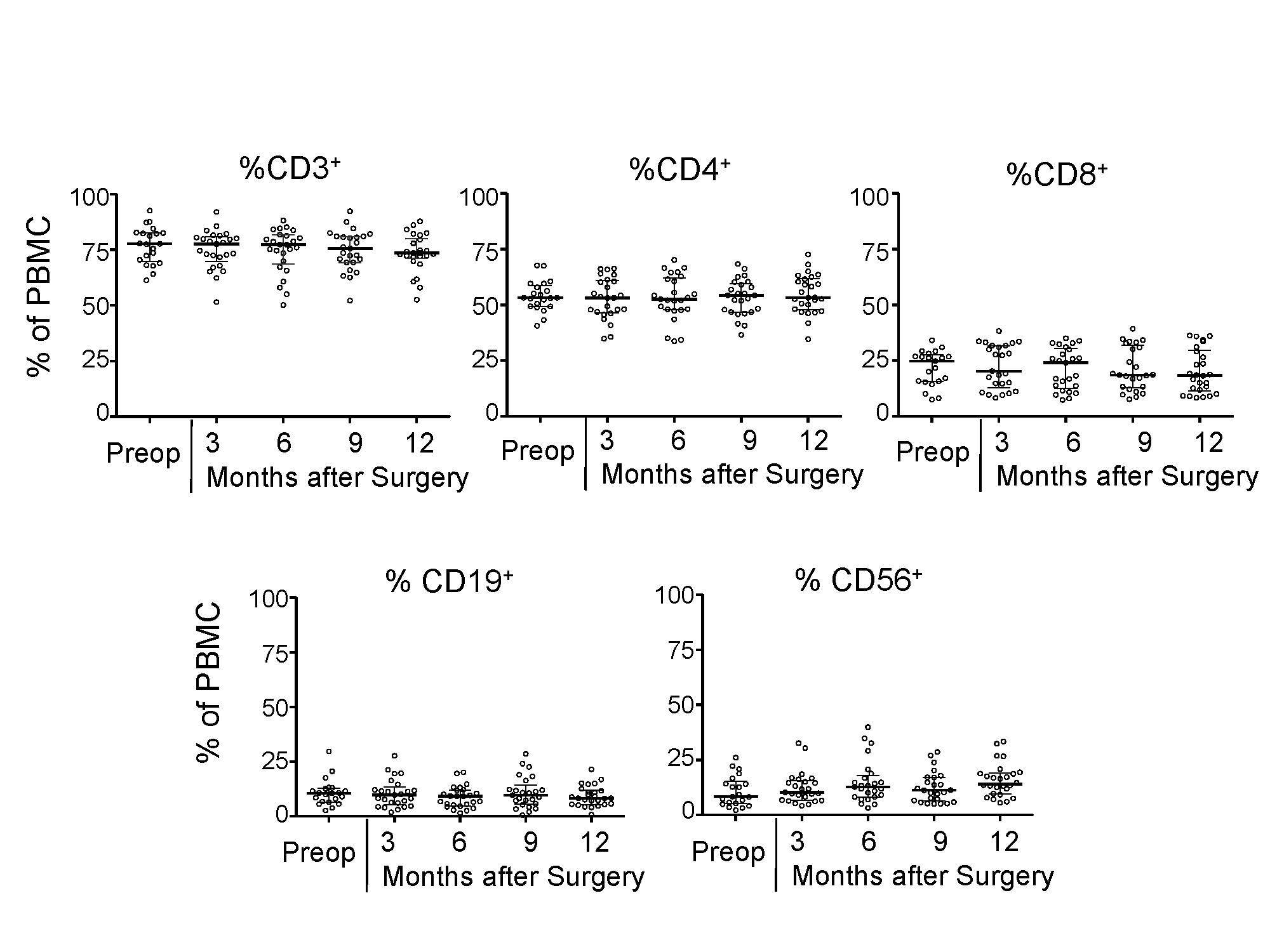

Supplement: Figure S2 — PBMC lymphocyte fractions from patients with stage I lung cancer. PBMC pellets were subjected to flow cytometry evaluating CD3, CD4, CD8, CD19 and CD56. Each data point represents an individual blood sample, while the horizontal bars represent the mean (+/−SEM). “Pre” indicates samples obtained prior to resection. (TIFF) [file pone.0064456.s002.tiff]
