# Supplementary material for: The Effect of Lung Cancer on Cytokine Expression in Peripheral Blood Mononuclear Cells
Source: PLoS One. 2013 Jun 6;8(6):e64456. doi: 10.1371/journal.pone.0064456 (PMC3675097; doi:10.1371/journal.pone.0064456)
Supplement: Table S1 — Primer and probe sets for qRT-PCR. (DOCX) [file pone.0064456.s004.docx]

**Supplemental Table 1. Primer and probe sets for qRT-PCR.**

| Gene symbol | 5’ Primer | 3’ Primer | Probe |
| --- | --- | --- | --- |
| ACTB | CCTGGCACCCAGCACAAT | GCCGATCCACACGGAGTACT | ATCAAGATCATTGCTCCTCCTGAGCGC |
| CCL3 | CCAGTTCTCTGCATCACTTTGCT | ATCTGCCGGGAGGTGTAGCT | TGACACGCCGACCGCCTGC |
| CXCL10 | TTCCTGCAAGCCAATTTTGTC | TCTTCTCACCCTTCTTTTTCATTGT | ACGTGTTGAGATCATTG |
| IL1β | CCCTAAACAGATGAAGTGCTCCTT | GGTGGTCGGAGATTCGTAGCT | CTGGACCTCTGCCCTCTGGATGGC |
| IL2Rα | CGTCCTGGGACAACCAATG | GGTGTCACTTGTTTCGTTGTGTTC | CAATGCACAAGCTC |
| IL8 | GCAGAGGGTTGTGGAGAAGTTT | TTGGATACCACAGAGAATGAATTTTT | TGAAGAGGGCTGAGAAT |
