# Supplementary material for: The Effect of Lung Cancer on Cytokine Expression in Peripheral Blood Mononuclear Cells
Source: PLoS One. 2013 Jun 6;8(6):e64456. doi: 10.1371/journal.pone.0064456 (PMC3675097; doi:10.1371/journal.pone.0064456)
Supplement: Table S2 — Demographic features of stage I lung cancer patients and control patients. (DOCX) [file pone.0064456.s005.docx]

**Supplemental Table 2: Demographic features of stage I lung cancer patients and control patients.**

|  | Cancer patients | Control patients | P value |
| --- | --- | --- | --- |
| Number | 62 | 32 |  |
| Mean age | 73 +/-1.2 | 64 +/-3.1 | 0.01^†^ |
| Female | 61% | 53% | 0.45^§^ |
| Never smoker | 12% | 47% |  |
| Former smoker | 80% | 50% | 0.001^§^ |
| Current smoker | 8% | 3% |  |

†Compared using a two-sample t-test

§Compared using Pearson’s χ^2^ test
